# A 3 base pair deletion in *TBX1* leads to reduced protein expression and transcriptional activity

**DOI:** 10.1038/srep44165

**Published:** 2017-03-08

**Authors:** Yuejuan Xu, Shaohai Fang, Erge Zhang, Tian Pu, Ruixue Cao, Qihua Fu, Fen Li, Sun Chen, Kun Sun, Rang Xu

**Affiliations:** 1 Department of Pediatric Cardiology, Xinhua Hospital, Affiliated to Shanghai Jiao Tong University School of Medicine, Shanghai 200092, China; 2 Scientific Research Center, Xinhua Hospital, Affiliated to Shanghai Jiao Tong University School of Medicine, Shanghai 200092, China; 3 Medical Laboratory, Shanghai Children’s Medical Center, Affiliated to Shanghai Jiaotong University School of Medicine, Shanghai 200127, China; 4 Department of Pediatric Cardiology, Shanghai Children’s Medical Center, Affiliated to Shanghai Jiaotong University School of Medicine, Shanghai 200127, China

## Abstract

Transcription factor TBX1 plays a pivotal role in heart development and has been implicated in 22q11.2 deletion syndrome. The structure of this protein has been elucidated, and several mutations have been identified that disrupt TBX1 localization, DNA/protein-binding, or mRNA expression. This study reports a mutation in the *TBX1* gene that leads to significantly reduced expression of the mutant protein. A total of 773 conotruncal heart defect patients and 516 unrelated healthy control individuals were enrolled, none of which harbored a 22q11.2 deletion or duplication. We identified a mutation, *c.303-305delGAA*, located in the third exon of *TBX1* that does not disrupt *TBX1* mRNA expression or DNA binding activity, but results in decreased TBX1 protein levels and transcriptional activity. Through protein degradation studies we demonstrated that TBX1 is degraded primarily in proteasomes. Although the *c.303-305delGAA* mutation leads to low levels of the mutant protein, we found that increased protein degradation was not the cause, and we hypothesize that an alternate mechanism, such as translational inhibition, may be the cause.

Congenital heart defects (CHDs) are the most common human birth defect, affecting nearly 1% of all live births^1^,^2^. Conotruncal heart defects (CTDs), also known as defects of cardiac outflow tracts, are the most common cyanotic CHDs, accounting for a fourth to a third of all CHDs. Common CTDs include the following: persistent truncus arteriosus (PTA); interrupted aortic arch (IAA); tetralogy of Fallot (TOF); pulmonary atresia with ventricular septal defect (PA/VSD); double outlet of right ventricle (DORV); and transposition of the great arteries (TGA). CTDs are usually serious and require surgical treatment, with patients often needing lifelong specialized cardiac care.

The pathogenesis of CTDs typically involves a combination of genetic and environmental factors. Most of the known causes of CHDs are sporadic genetic changes, either mutations or focal copy-number DNA variations. Small chromosomal abnormalities, such as microdeletions of 22q11.2 and 8p23, have been implicated in the pathogenesis of CTDs. Though the molecular pathways regulating the complex development of the embryonic heart have only been partly elucidated, a number of genes are associated with cardiac malformations, some of which are linked to specific defects.

TBX1 is a member of the phylogenetically conserved T-box transcription factor family and has been hypothesized to be involved in the pathogenesis of CTDs. The *TBX1* gene maps to the critical 1.5 Mb deletion region of 22q11.2. The loss of TBX1 is recognized as being largely responsible for the 22q11.2 deletion syndrome (22q11DS), the most common deletion syndrome, which also has the most extensive symptoms among CTDs. Experiments with mouse models showed that *TBX1* heterozygous and homozygous null mutants exhibited CTDs and other phenotypes similar to those observed in 22q11.2 DS patients^3^. Further studies elucidated that TBX1 plays a crucial role in normal pharyngeal and heart development and is involved in regulating the migration, proliferation and differentiation of cardiovascular progenitor cells during heart formation^4^,^5^,^6^,^7^.

Support for the importance of this gene in humans came from patients exhibiting a 22q11DS phenotype that lacked the deletion of *TBX1* but instead had a mutation in *TBX1*. Mutational analyses in nonsyndromic CTD patients also identified two loss of function TBX1 mutants^8^,^9^. Here, we report a mutation (*c.303-305delGAA*), which caused the reduced expression of the TBX1 protein in a patient with TOF.

## Results

### Identification of a homozygous *TBX1* mutation (*c.303-305delGAA*) in a TOF patient

**Figure 1:**
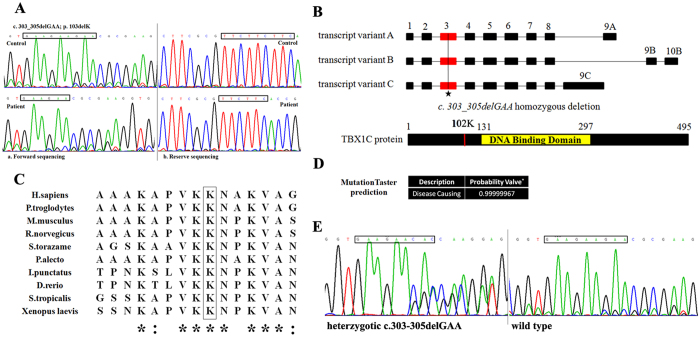
Characteristics of the mutation *c.303-305delGAA* in *TBX1*. (**A**) Chromatograms of the homozygous mutation found in the TOF patient of cohort 1; (**B**) Structural representations of the mutation in *TBX1* gene and protein; (**C**) Homology analysis of the 102 K position of TBX1 protein across different species; (**D**) the prediction result of MutationTaster. (*The probability value is the probability of the prediction, and a value close to 1 indicates a high ‘security’ of the prediction). (**E**) Chromatograms of the heterozygous *c.303-305delGAA* mutation found in cohort 2.

No copy number variants of 22q11.2 or any other chromosome region were identified in any of the 176 patients in cohort 1 using the SALSA P250-A1 kit (data not shown). Out of the 176 CTD cohort 1 patients, we identified a *TBX1* nucleotide alteration, localized in exon 3, present in a 24-month-old boy suffering from TOF. The patient is homozygous for *c.303-305delGAA* (Fig. 1A). Multiplex ligation-dependent probe amplification (MLPA) results confirmed that the 22q11.2 deletion was not present in the patient. The patient’s face presents as normal, and he had no other extracardiac malformations. The patient’s parents were in a non-consanguineous marriage but have since divorced. Unfortunately, because the parents refused to participate in this study, we could not identify the source of this mutation.

The *c.303-305delGAA* mutation causes a deletion of TBX1 codon 102, which encodes lysine (p.102delK) (Fig. 1B). This mutation was not detected in the cohort 1 healthy controls. A Clustal Omega analysis revealed that the TBX1 102 K residue is highly conserved among different species (Fig. 1C), and the mutation is predicted to be “disease causing” by MutationTaster software (Fig. 1D).

To further evaluate the frequency of the *c.303-305delGAA* mutation in the Chinese Han population, we sequenced the locus in cohort 2 individuals. Cohort 2 consisted of 594 CTD patients who were negative for the 22q11.2 deletion or duplication^10^, as well as 377 healthy control individuals. In total, 14 out of 594 CTD patients (1.2%) were found to harbor a heterozygous *c.303-305delGAA* mutation (Fig. 1E, Table 1). In comparison, the same heterozygous mutation was detected in 2 healthy controls (0.3%). Considering these findings, we speculated that at least one allele of the homozygous *c.303-305delGAA* mutation detected in the proband might have been transmitted from his seemingly healthy parents. However, we could not verify this hypothesis due to the noncooperation of these individuals. The genotype for the mutation was shown to be in Hardy-Weinberg equilibrium (*p* > 0.05) (data not shown). The frequency of the *c.303-305delGAA* mutation in CTD patients differed from that of healthy controls (1.2% vs. 0.3%; *p* = 0.037) (Table 2). This difference implies that the *TBX1* heterozygous *c.303-305delGAA* mutation may have an impact on the pathogenesis of CTDs.

### The *c.303-305delGAA* mutation does not perturb TBX1 protein location

**Figure 2:**
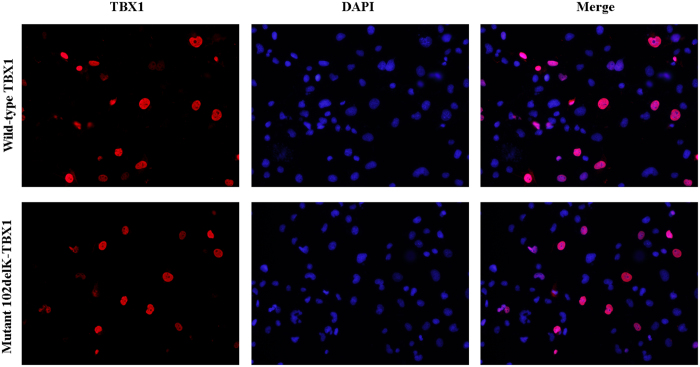
Subcellular localization of wild-type and mutant TBX1 in transiently transfected COS7 cells. Cells were co-stained with 4,6-diamino-2-phenylindole (DAPI) to illustrate nuclei. Wild-type TBX1 and the p.102delK mutant both localize to the nucleus.

Immunocytochemistry demonstrated that the p.102delK mutated protein (TBX1^102delK^) protein localized to the nucleus, indicating that the mutation does not perturb protein location (Fig. 2).

### The *c.303-305delGAA* mutation causes decreased TBX1 protein levels and reduced transcriptional activity

**Figure 3:**
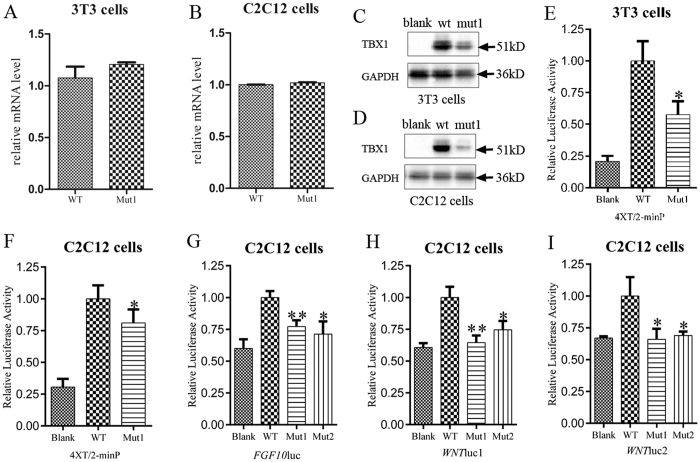
The mutant protein TBX1^102delK^ showed decreased expression level, and lost transcriptional activity. (**A**,**B**) Real-time qPCRs show the mRNA level of the mutant *TBX1*^*c.303_305delGAA*^ was similar with that of the wild-type *TBX1* in both 3T3 and C2C12 cells. (**C**,**D**) Western blots show the protein level of the mutant TBX1^102delK^ and wild-type TBX1. The mutant showed reduced protein level compared with the wild-type in both 3T3 and C2C12 cells. The full-length blots are presented in the Supplementary Figure S1 and S2, respectively. (**E**,**F**) show that the 3T3 or C2C12 cells were co-transfected with the 4XT/2-minP reporter and either a pcDNA3.1(+) control vector (Blank), the TBX1 wild-type construct (WT), or the mutant *TBX1*^*c.303_305delGAA*^ construct (Mut1). The results were normalized for transfection efficiency to a co-transfected pGL4.74[hRluc/TK] vector. The TBX1^102delK^ mutant showed significantly reduced transcription activity compared with the wild-type protein in both 3T3 (reduced 42.4% vs. *TBX1*^*wt*^, *p* = 0.0112) and C2C12 cells (reduced 19.9% vs. *TBX1*^*wt*^, *p* = 0.0164). (**G**) Shows the C2C12 cells were co-transfected with the *FGF10*luc reporter and either the blank, the wild-type, or the mutant constructs (Mut1 = TBX1^102delK^ and Mut2 = TBX1^E129K^). The results were normalized for transfection efficiency to a co-transfected pGL-TK vector. The TBX1^102delK^ and TBX1^E129K^ mutants all showed significantly reduced transcription activity compared with the wild-type protein (reduced 22.9% vs. *TBX1*^*wt*^, *p* = 0.0050 and reduced 28.7% vs. *TBX1*^*wt*^, *p* = 0.0112, respectively). (**H**) shows that significantly reduced transcriptional activity of the TBX1^102delK^ and TBX1^E129K^ mutant proteins on the *WNT*luc1 reporter compared with the wild-type protein in C2C12 cells (reduced 35.5% vs. *TBX1*^*wt*^, *p* = 0.0037 and reduced 25.4% vs. *TBX1*^*wt*^, *p* = 0.0156, respectively). (**I**) shows that the transcription activity of the two mutant proteins also decreased significantly on the *WNT*luc2 reporter in C2C12 cells (reduced 34% vs. *TBX1*^*wt*^, *p* = 0.0259 and reduced 31% vs. *TBX1*^*wt*^, *p* = 0.0242, respectively). The results are all shown as the mean ± SEM of three independent experiments performed in triplicate. *p < 0.05; **p < 0.01; blank: pcDNA3.1(+) vector; WT: wild-type TBX1; mut1: the TBX1^102delK^ mutant; mut2: the TBX1^E129K^ mutant.

Wild-type and mutant TBX1 expression plasmids (*TBX1*^*wt*^and *TBX1*^*c.303-305delGAA*^, respectively) were generated and separately transfected into C2C12 and NIH3T3 cells. We observed that wild-type and mutant *TBX1* mRNA levels did not differ in either cell lineage (Fig. 3A,B). Interestingly, in both NIH3T3 and C2C12 cells, expression of the TBX1^102delK^ mutant protein was greatly reduced compared to the wild-type protein, as assessed by western blot (Fig. 3C,D) (reduced 62.1% vs. *TBX1*^*wt*^ in 3T3 cells and 79.4% in C2C12 cells, Figures S1 and S2). In luciferase assays, the mutant protein also produced significantly reduced transcriptional activity compared with the wild-type protein using the 4XT/2-minP reporter^9^,^11^ in both NIH3T3 (reduced 42.4% vs. *TBX1*^*wt*^, *p* = 0.0112) and C2C12 cells (reduced 19.9% vs. *TBX1*^*wt*^, *p* = 0.0164) (Fig. 3E,F).

Wnt5a and Fgf10 have been reported as transcriptional targets of TBX1^12^,^13^. To further validate the effect of the mutation, we constructed the *WNTluc1*, *WNTluc2* and *FGF10luc* reporter plasmids as described by Chen, L. *et al*.^12^ and Agarwal, P. *et al*.^13^, respectively. Luciferase assays were conducted in the C2C12 mouse myoblast cell line. *TBX1*^*wt*^or *TBX1*^*c.303-305delGAA*^expression vectors were co-transfected with the *WNTluc1*, *WNTluc2* or *FGF10luc* reporter plasmids. A construct expressing a second TBX1 mutant protein (TBX1^E129K^), which carries a mutation within the T-box binding, protein-DNA interaction domain^9^, was also cotransfected. Both the TBX1^102delK^ and TBX1^E129K^ mutant proteins exhibited partially reduced transcriptional activity for the *FGF10* promoter (reduced 22.9% vs. *TBX1*^*wt*^, *p* = 0.0050 and reduced 28.7% vs. *TBX1*^*wt*^, *p* = 0.0112, respectively) (Fig. 3G) and reduced transcriptional activity for the two *WNT5A* reporters in C2C12 cells (reduced 35.5% vs. *TBX1*^*wt*^, *p* = 0.0037 and reduced 25.4% vs. *TBX1*^*wt*^, *p* = 0.0156 for the *WNT*luc1, respectively; reduced 34% vs. *TBX1*^*wt*^, *p* = 0.0259 and reduced 31% vs. *TBX1*^*wt*^, *p* = 0.0242 for the *WNT*luc2, respectively) (Fig. 3H,I). Unlike the E129K mutation, the p.102delK mutation is not located in the TBX1 T-box binding domain. The reduced luciferase activity resulting from the p.102delK mutation is in agreement with the observed reduction in TBX1^102delK^ protein levels compared to the wild-type protein. We speculated that the low expression of the mutant TBX1^102delK^ protein resulted in its loss-of-function.

### The *c.303-305delGAA* mutation does not impair TBX1 DNA binding activity

**Figure 4:**
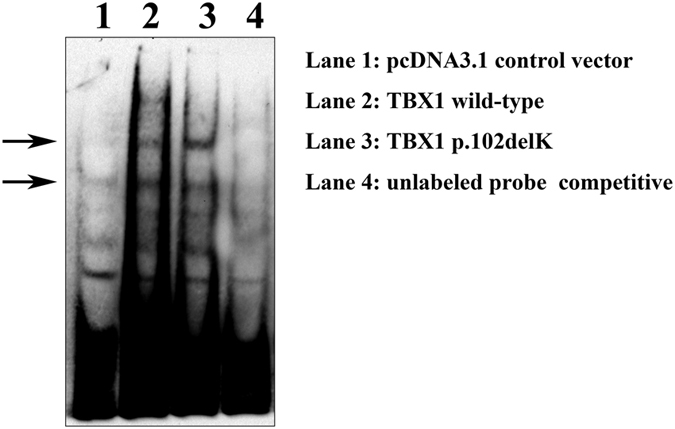
Electrophoretic mobility shift assay to study DNA-binding activity of wild-type and mutant TBX1. DNA binding assay show that both the wild-type and p.102delK mutant TBX1 could bind to DNA (lane 2 and 3). Specificity of the binding is confirmed by nuclear protein of HEK293T cell transfected with the pcDNA3.1(+) control vector (lane 1) and the addition of unlabled probe (100X) (lane 4). The full-length image is presented in the Supplementary Figure S3.

The T-box domain of TBX1 is known to directly interact with cognate sequences (T-sites) on genomic DNA^11^. Because reduced transcriptional activation could also result from the impaired DNA binding of TBX1^102delK^, we tested whether the mutation affected DNA binding via EMSA (Fig. 4). HEK293T cells were transfected with the pcDNA3.1(+) control vector (control), the wild-type *TBX1*^*wt*^ construct (WT), or the mutant *TBX1*^*c.303-305delGAA*^ construct (Mut). To ensure a sufficient amount of protein was produced, MG-132 was added to the culture medium 8 hours prior to harvesting cells, from which nuclear proteins were prepared for the DNA binding assays. The EMSA results revealed that the TBX1 wild-type and TBX1^102delK^ mutant proteins directly bound the 3’-biotin labeled probes, suggesting that the binding of the mutant protein to T-box DNA was not impaired.

### The *c.303-305delGAA* mutation does not accelerate protein degradation

**Figure 5:**
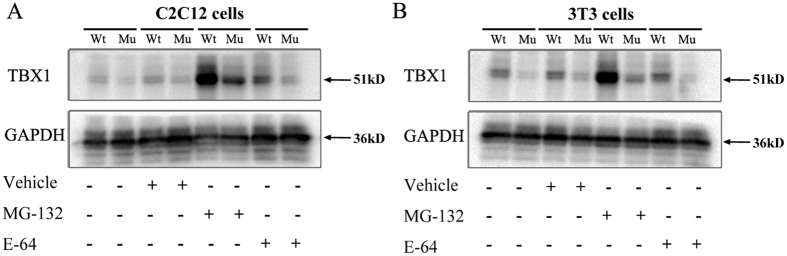
Proteasome inhibitor MG-132 increases both wild-type and mutant TBX1 protein level. C2C12 cells and 3T3 cells were transfected with equal concentrations of TBX1-encoding plasmids (the wild-type or the mutant 102delK) and treated with 20uM MG-132 or 10uM E-64 for 8 hours. The TBX1 levels were determined by Western blot (A: C2C12 cells; B: 3T3 cells). GAPDH was used as an internal control. n = 3. Wt: wild-type; Mut: the TBX1^102delK^ mutant; vehicle: DMSO. The full-length image is presented in the Supplementary Figure S4.

Although we observed reduced levels of the TBX1^102delK^ mutant protein, mRNA levels in the mutant and wild-type were similar. We speculated whether the decreased expression of the TBX1^102delK^ protein resulted from instability and increased degradation. To evaluate TBX1^102delK^ degradation, we treated the *TBX1*^*c.303-305delGAA*^ transfected 3T3 and C2C12 cells with MG-132, an inhibitor of 26S proteasomal degradation. In the presence of MG-132, the observed protein levels of both the wild-type and mutant TBX1 increased dramatically in both 3T3 and C2C12 cells. However, TBX1^102delK^ mutant protein levels remained lower than the wild-type protein. We also treated the transfected cells with the lysosomal inhibitor E-64, the protein levels of both the wild-type and mutant TBX1 increased slightly, and the mutant protein also remained lower than the wild-type (Fig. 5). These combined data suggest that TBX1 is degraded primarily in proteasomes. However, the significantly decreased TBX1^102delK^ protein levels were not caused by increased degradation.

## Discussion

CTDs are caused by defects in the development of the outflow tract, branchial arch, and arteries of the heart during organogenesis. Development of the pharyngeal arch arteries and the second heart field (SHF) contributes to the formation of the outflow tract during heart development^14^,^15^. The *TBX1* gene, which belongs to an evolutionarily conserved T-box family of transcription factors, is expressed in the second heart field and throughout the pharyngeal arches. *TBX1* appears to regulate the migration, proliferation, and differentiation of cardiovascular progenitor cells during heart development, and its expression is precisely regulated during these processes^16^,^17^,^18^,^19^. Previous studies in mice have shown that *TBX1* acts in a dose-dependent manner, and the progressive dosage reduction of *TBX1* mRNA is associated with a non-linear increase in phenotypic severity^20^,^21^. On the other hand, the overexpression of *TBX1* also results in the malformation of the outflow tract^21^,^22^.

TBX1 has several functional domains, including a DNA binding domain, a transcriptional activity domain, and a nuclear localization signaling domain^23^. Several mutations of the human *TBX1* gene have been identified, which disturb the localization, DNA/protein-binding, or expression of the TBX1 protein^24^,^25^,^26^. The first reported *TBX1* mutation (*c.1223delC*), as well as a second (*c.1320-1342del23*), damage the TBX1 nuclear localization signal and prevent the mutant protein from transport into the nucleus^24^,^27^. Other missense mutations within the DNA-binding and transcriptional activity domain (such as H194Q, F148Y, and E129K) are predicted to affect the protein-protein or DNA-protein interactions and result in gain or loss of functions^9^,^24^,^25^. A mutation upstream of the DNA-binding domain (*c.129-185del57*) results in a less stable TBX1 mutant protein^26^. Lastly, a mutation outside of the coding sequence, within the 5′UTR (*c.-39C* > *T*), is predicted to increase the production of *TBX1* mRNA^28^.

In the present study, we carried out a comprehensive analysis of *TBX1* mutations in Chinese patients with CTDs. Our survey of *TBX1* exons and flanking intron sequences in these patients identified a mutation, *c.303-305delGAA*. This mutation results in the deletion of a lysine residue at position 102 of the protein, outside of both the NLS domain and the transcriptional activity domain. The mutant protein localized to the nucleus (Fig. 2); however, it showed reduced transcriptional activity (*p* < 0.05) (Fig. 3E–I). Western blot assays showed that mutant protein levels were significantly decreased compared to those of the wild-type protein (Fig. 3C,D). DNA binding assays showed that both the wild-type and mutant proteins could bind DNA (Fig. 4), suggesting that the reduced TBX1^102delK^ protein accounted for decreased transcriptional activity. These results suggest that dosage reduction of the mutant TBX1^102delK^ protein is the cause of the cardiac phenotype in the patient carrying the homozygous *c.303-305delGAA* mutation. The heterozygous *TBX1c.303-305delGAA* mutation was detected in 14 out of 594 (1.2%) CTD patients as well as 2 out of 377 (0.3%) healthy controls in our cohort 2. The frequency of the *c.303-305delGAA* mutation in CTD patients differed from that of healthy controls (*p* = 0.037) (Table 2), suggesting that the heterozygous *TBX1c.303-305delGAA* mutation may impact the pathogenesis of CTDs. However, further studies are needed to exclude mutations in other cardiac transcription factors (such as GATA4, NKX2.5, and TBX5) as the cause of CTDs present in these patients.

Proteasomes degrade misfolded or nonfunctional proteins to recycle intracellular amino acids. The proteasome is a multisubunit complex that degrades various cellular proteins that are attached to a multiubiquitin chains^29^. The ubiquitin proteasome system is the major pathway for intracellular protein degradation. MG-132, which acts as a blocker in ubiquitin-proteasome pathway, is involved in >80% of intracellular protein degradation^30^. The *TBX1c.303-305delGAA* mutation results in small but detectable amounts of TBX1^102delK^ protein. To investigate the possible altered proteasome degradation of TBX1^102delK^ compared to the wild-type protein, we utilized MG-132 to block ubiquitination dependent degradation. The inhibition of proteasome activity elevated wild-type and mutant TBX1 protein levels in C2C12 and 3T3 cells. However, MG-132 did not block the observed decrease in mutant TBX1^102delK^ protein levels. Additionally, slightly increase in TBX1 protein expression (both the wild-type and mutant) was observed after the addition of the lysosomal inhibitor E-64. These results suggest that the proteasome, rather than the lysosome, is involved in the degradation of wild-type TBX1 protein. However, the *c.303-305delGAA* mutation did not lead to an increase in the TBX1 degradation. It is possible that other mechanisms may be involved in the decrease of the mutant TBX1^102delK^ protein, such as the inhibition of protein translation. The *c.303-305delGAA* mutation may alter the secondary structure of mRNA, possibly disrupting TBX1 translation. Further investigation is required to confirm this hypothesis.

## Conclusion

In this study, we reported a *c.303-305delGAA* mutation in the human gene *TBX1*, which leads to significantly reduced expression and transcriptional activity of the mutant protein. Our data suggest that the ubiquitin proteasome pathway is crucial for TBX1 protein degradation. However, the *c.303-305delGAA* mutation does not cause altered protein degradation. It is possible that other mechanisms, such as translational inhibition, may have resulted in the observed decrease of TBX1^102delK^ mutant protein levels.

## Materials and Methods

### Ethics statement

All assessments were done with the approval of the Medical Ethics Committee of Xinhua hospital and shanghai Children Medical Center. And all experiments were carried out in accordance with the approved guidelines. Fully written informed consent was obtained from all participants (or their parents if children were too young to consent by themselves).

#### Study population

For the screening of *TBX1* mutation, patients were recruited in Xinhua hospital from March 2012 to May 2013. Clinical records including echocardiography and angiocardiography were reviewed by Pediatric cardiologists before recruitment, and patients who had one or more extracardiac anomalies were excluded. 176 (69 female, 107 male; median age 2.53 years) unrelated CTDs patients were enrolled (patients in the cohort 1). They included 72 TOF, 54 DORV, 28 PA/VSD and 22 TGA. All of the subjects were of Han ethnicity. 139 unrelated physically and mentally healthy Chinese children (57 female, 83 male; all ethnically Han) were included as normal controls. The median age was 7.43 years (healthy control in the cohort 1). The normal cardiac morphology of these controls was confirmed by transthoracic echocardiography at the Xinhua hostipal. DNA was extracted from the blood samples using the QIAamp DNA Blood Mini Kit (Qiagen, Duesseldorf, Germany).

In order to detect the frequency of the gene mutation, 594 CTDs patients (including 234 TOF, 99 DORV, 98 PA/VSD, 10 PTA, 46 single atria/single ventricle, 13 PA/IVS (intact ventricular septum), 92 D-TGA and 13 IAA) were enrolled from November 2011 to January 2014 in shanghai Children’s Medical Center (patients in the cohort 2)^10^. All of the patients have been negative to 22q11.2 deletion or duplication, detected by CNVplex^10^. All subjects were unrelated and of Han ethnicity. The median age of them at the time of diagnosis was 10 months with a range of 3 days to 18 years. A cardiologist confirmed the CHD diagnosis for all patients by reviewing and evaluating patient history, physical examinations, and medical records^10^. 377 unrelated healthy individuals (median age was 3.2 years), free of CHD, of Han ethnicity were also enrolled as controls (healthy control in the cohort 2). The normal cardiac morphology of these controls was confirmed by transthoracic echocardiography at shanghai Children Medical Center. DNA was extracted from the blood samples using the standard protocols.

### Multiplex ligation-dependent probe amplification (MLPA)

MLPA was performed as previously described^31^. The SALSA P250-A1 MLPA-DiGeorge syndrome test kit (MRC-Holland, Amsterdam, the Netherlands) was used to detect of the 22q11.2 deletion in patients of cohort 1. 100ng of DNA was used following the manufacturer’s protocols. The PCR products were sequenced by an ABI 3130XL Genetic Analyzer (Applied Biosystems, Foster City, CA, USA). The raw data were analyzed using the Coffalyse. Net software (MRC-Holland). A threshold of signal intensity change of <0.75 was used to identify potential deletions, and a threshold of >1.3 was used to identify potential duplications.

### Gene sequencing and mutation characterization

Mutational screening was performed using direct sequence analysis of all *TBX1* exons and flanking intron sequences in patients and control of cohort 1. PCR primers (provide on request) were designed based on the corresponding genomic regions available on the Genbank database (NG_009229.10) using Primers3 software (http://bioinfo.ut.ee/primer3/). PCR reactions were performed on the Veriti^®^ 96-Well Thermal Cycler using 100 ng of genomic DNA in a volume of 25 μl containing 1X PCR buffer, 1.5 mM MgCl_2_, 250 μM dNTPs, 0.200 μM of forward and reverse primer, and 2.5U TaKaRa Ex Taq (Takara, Shiga, Japan). PCR products were purified using Exo/SAP treatment and sequenced using an ABI 3730 DNA Analyzer (Life Techonlogies, USA) by Sanger sequencing, and sequence traces were analyzed using the BLAST program (http://blast.ncbi.nlm.nih.gov/Blast.cgi) and the Mutation Surveyor V4.0.5 software (SoftGenetics Inc., State College, PA, USA).

For the mutations detected in the cohort 1, the frequency of the mutation was further evaluated in the cohort 2 (594 CTDs patients and 377 healthy controls) using the Sanger sequencing.

### Plasmid Constructs

The human *TBX1* isoform C cDNA clone was purchased from OriGene. The TBX1 wild-type plasmid was generated by subcloning the *TBX1* cDNA into the expression vector pcDNA3.1(+) between the KpnI and XhoI sites, as reported previously^9^. The mutant plasmid was generated by site-directed mutagenesis using the QuikChange Site-Directed Mutagenesis Kit (Agilent Technologies, Santa Clara, CA, USA), pcDNA3.1-TBX1 wild-type was used as template.

To construct the luciferase reporter plasmid, four conserved “ATTTCACACCT” T-half sites oriented head to tail, was synthesized and cloned into the KpnI−HindIII sites in the pGL4.25[luc2CP/minP] plasmid (Promega, Madison, Wisconsin, USA) to generate the 4XT/2-minP reporter construct^9^,^11^. The sequence and orientation of the luciferase reporter were verified by DNA sequencing. Wnt5a has been reported as a transcriptional target of Tbx1^12^, so we cloned the two DNA segments containing the conserved T-box binding sites (TBEs) in the intron 1–2 and 3′-UTR of human *WNT5A* gene and inserted into the pGL3-promoter plasmid (Promega, Madison, Wisconsin, USA). The two luciferase reporters were referred as to *WNT*luc1 and *WNT*luc2 reporter, respectively. An *FGF10*luc reporter was also constructed according to the described by Agarwal P *et al*.^13^. *FGF10* genomic sequence was obtained from the GenBank (http://www.ncbi.nlm.nih.gov). A fragment comprising 4.5 kb upstream of the coding region (−4.3 kb/ + 176 bp) of *FGF10* was amplified from DNA of a healthy individual and inserted into the MluI/SmaI sites of the pGL3-Basic vector (Promega, Madison, Wisconsin, USA) to generate the *FGF10*luc reporter construct.

### Cell Culture and Transient Transfection

HEK293T cells, COS7 cells, NIH3T3 cells and C2C12 cells were cultured in Dulbecco’s modified Eagle medium (DMEM) (Invitrogen, California, USA) supplemented with 10% fetal bovine serum (FBS) (Invitrogen, California, USA), 100 μg/ml penicillin, and 100 μg/ml streptomycin in a humidified incubator at 37 °C with 5% CO_2_. For transient transfection, cells were cultured in 6-well plates until 70–80% confluent and transfected with FuGENE HD (Promega, Madison, Wisconsin, USA) according to the manufacturer’s protocol. When required, one day after transfection, the proteasome inhibitor MG-132 (Sigma, St. Louis, MO, USA) or the cysteine protease inhibitor E-64 (Sigma, St. Louis, MO, USA) were added to the culture medium for 8 hours at a concentration of 20 uM or 10 uM, respectively. MG-132 and E-64 were all dissolved in Dimethyl sulfoxide (DMSO). Whole cell lysate was used in Western blots analysis.

### RNA isolation and Real-time quantitative reverse-transcript PCR

The same amount of wild-type and mutant TBX1 vectors were separately transfected into NIH3T3 and C2C12 cells. 24 hours after transfection, total RNA was extracted using TRIzol reagent (Invitrogen, Carsbad, CA, USA) according to the manufacturer’s instrunctions. The purity and quantity of RNA were measured using Nanodrop (ND-2000, Thermo Fisher Scientific Inc., Waltham, MA, USA). 1.0 ug of total RNA in a volume of 20ul was reverse transcribed to cDNA using the PrimeScript^TM^ RT Master Mix Kit (Takara, Shiga, Japan). Complementary DNA-samples were diluted 5-fold and used as a template in each reaction. The real-time quantitative PCR was performed using the SYBP^®^ Premix ExTaq^TM^ Kit (Takara, Shiga, Japan) according to the manufacturer’s instructions. The reactions were in a final volume of 20 ul and carried out on the ABI Prism^®^ 7900 HT Sequence Detection System (Applied biosystems, Foster City, CA, USA). Glyceraldehyde-3-phosphate dehydrogenase (GAPDH) was used as the internal control. The PCR primer sequences were as follows: TBX1: 5′-ATGTGGACCCACGCAAAGATAG-3′ (forward) and 5′-GGCAATCTTGAGCTGCGTGA-3′ (reverse), and GAPDH: 5′-TGCCTT CTTGCCTCTTGTCTCT-3′ (forward) and 5′-TTTCTTCCATTCTGTCTTCCACTC-3′ (reverse). Triplicate measurements were performed for all reactions. The melting curses were analyzed automatically by collecting of the flurescence signals. Fold change was calculated using the 2^−△△Ct^ method^32^, with GAPDH mRNA expression levels used for normalization.

### Immunocytochemistry

COS7 cells were seeded on glass cover slips and transfected with the *TBX1*^*wt*^plasmid and the mutant *TBX1*^*c.303_305delGAA*^plasmid, respectively. 48 hours after transfection, the cells were washed three times with phosphate-buffered saline (PBS), fixed for 20 min in 4% paraformaldehyde, washed and permeabilized in 0.2% Triton X-100/PBS. Later the cells were blocked with 5% bovine serum albumin in PBS at room temperature for 1 hour. Subsequently the cells were incubated with anti-TBX1 antibody (1:100 dilution) (invitrogen, California, USA) overnight at 4 °C, and followed by a further wash in PBS. The Rhodamine -conjugated anti-rabbit secondary antibody (1:1000 dilution) (Life Technologies) was then applied for 2 hours at room temperature. At last, the cells were washed and treated with 0.5 μg/ml DAPI (Roche, Indianapolis, IN, USA) to visualize the nucleus. Positive staining was visualized with a fluorescence microscope (Olympus, Tokyo, Japan).

### Western Blot analysis

Total proteins were extracted from the transfected cells treated with MG132, E62 or DMSO. Protein concentration was quantified using the Micro BCA^TM^ Protein Assay Kit (Thermo Scientific, Rockford, IL, USA). Then, 30 μg proteins were separated by 10% SDS-PAGE and transferred onto nitrocellulose membranes (Millipore, Billerica, MA, USA). After that, the membranes were blocked in 5% skim milk for 2 hours at room temperature (RT) and then incubated with primary antibodies overnight at 4 °C. The primary antibody used were anti-TBX1 (1:400 dilution) (invitrogen, California, USA) and anti-GAPDH (1:1000 dilution) (Proteintech, Chicago, IL, USA). Subsequent to the removal of the primary antibody by washing, horseradish peroxidase (HRP) – conjugated antibodies (1:1000 dilution) (Proteintech, Chicago, IL, USA) were then incubated with membranes for 2 hours at RT. The membranes were washed again, and detected using enhanced chemiluminescence (ECL) reagents (Millipore, Billerica, MA, USA) according to the manufacturer’s instrunctions. Bands were visualized with a chemiluminescence detection system (BioRad, Philadelphia, PA, USA) and analyzed using the Imagelab program (BioRad, Philadelphia, PA, USA). To analyze protein degradation, both transfected C2C12 and 3T3 cells were treated with 20 uM MG-132 or 10 uM E-64 for 8 h.

### Dual luciferase reporter assays

Plasmids including the wild-type or mutant TBX1 vector, the firefly luciferase reporter and the corresponding renilla luciferase reporter were co-transfected into NIH3T3 cells and/or C2C12 cells. 40 hours after transfection, cells were harvested and lysed in passive lysis buffer (Promega, Madison, WI, USA). Firely and Renilla luciferase activity were measured using the Dual Luciferase Kit (Promega, Madison, Wisconsin, USA) and the Centro XS^3^ LB 960 Microplate Luminometer (Berthold, Bad Wildbad, Germany) according to the manufacturer’s recommended protocol. The transfection efficiency was normalized to paired Renilla luciferase activity. The results represent the mean ± SEM of three independent experiments performed in triplicate.

### Electrophoretic mobility shift assays (EMSA)

HEK293T cells were cultured in 6-well plate and transfected with the pcDNA3.1 plasmid, *TBX1*^*wt*^plasmid and the mutant *TBX1*^*c.303_305delGAA*^plasmid, respectively. To ensure a sufficient amount of protein was produced, MG-132 was added to the culture medium for 8 hours before harvest. Nuclear extracts were prepared using nuclear protein extraction kit (Pierce Biotechnology, Rokford, IL, USA). The sequence of the palindromic oligonucleotides probe was 5′-CTAGATTTCACACCTAGGTGT-3′^32^. The probes were synthesized and labeled with biotin in the 3′-end. Unlabeled oligonucleotides with identical sequences were used as competitors. The EMSA was performed using the LightShift Chemiluminescent EMSA kit (Pierce Biotechnology), following the manufacturers’ protocol. Brefly, 20 ug of nuclear protein sample were incubated with 0.02 pmol of 3′-labeled biotin probes, 10 X binding buffer, 50% Glycerol, 1% NP-40, 100 mM MgCl_2_, 1 ug/ul poly(dI-dC), 200 mM EDTA, 1 M KCl in the presence or absence of competitor oligonuleotides. All reactions were carried out at room temperature for 20 min. The products were then separated by 6.5% nondenaturing polyacrylamide gels in 0.5 x TEB. The DNA-protein complex was electrotransferred to a piece of nylon membrane (Pierce), and the signal was detected by the chemiuminescent Nucleic Acid Detection Module kit (Pierce).

### Statistical analysis

The reported values correspond to the means ± SEM obtained from three independent experiments. The independent-samples t-test was used to determine statistical significance of unpaired samples. *P* < 0.05 was regarded as statistically significant. The Hardy-Weinberg equilibrium (HWE) and allele frequencies were assessed at the single nucleotide polymorphism (SNP) locus using the χ^2^ and Fisher’s exact test. HWE_*P* value > 0.05 was considered as a statistically insignificant deviation from the equilibrium. The 2-sided statistical tests were considered significant with a level of *P* ≤ 0.05. All statistical analysis in our study was carried out with SPSS 22.0 (SPSS Inc. Chicago, IL, USA).

## Additional Information

**How to cite this article:** Xu, Y. *et al*. A 3 base pair deletion in *TBX1* leads to reduced protein expression and transcriptional activity. *Sci. Rep.* **7**, 44165; doi: 10.1038/srep44165 (2017).

**Publisher's note:** Springer Nature remains neutral with regard to jurisdictional claims in published maps and institutional affiliations.

## Supplementary Material

Supplementary Information

## Figures and Tables

**Table 1:** Detailed information of the 14 CTDs patients with heterozygous *c.303-305delGAA* in *TBX1*.

Sample No.	*Age (m)	Gender	Cardiac Defects
A038	12	M	TGA/ASD(II)/TR
A057	8	M	TGA/VSD/ASD(II)/PS
A059	60	F	TGA/VSD/PS
A070	1	M	TGA/VSD/ASD(II)/PDA
F089	8	F	TOF/PFO
F106	12	M	TOF/ASD(II)/VSD/LSVC
F189	5	M	TOF/ASD(II)/PDA
F199	18	M	TOF/PFO/PDA
F203	5	F	TOF/ASD(II)
S030	1	M	TGA/SV/PS/ASD(II)
V012	96	F	DORV/VSD/PS/LSVC
V029	4	F	DORV/VSD/ASD(II)
V030	16	F	DORV/VSD/PS
V031	5	M	DORV/VSD/ASD(II)

Abbreviations: *age in months at the point of examination; m = months; ASD (II) = secondary atrial septal defect; VSD = ventricular septal defect; TR = tricuspid regurgitation; PS = pulmonary stenosis; PDA = patent ductus arteriosus; PFO = Patent Foramen Ovale; LSVC = persistent left superiror vena cava; SV = single ventricle.

**Table 2:** Allele frequency of *c.303-305delGAA* of *TBX1* in cohort 2 (CTDs patients = 594, control = 377).

	Frequency of SNP ((%)n)	
	*c.303-305delGAA*	Non del
patients	1.2% (14)	98.8% (1174)
control	0.3% (2)	99.7% (752)
χ^2^	4.708	
*P	0.037	

*P-value was calculated using Fisher’s exact test.
