# Supplementary material for: A 3 base pair deletion in TBX1 leads to reduced protein expression and transcriptional activity
Source: Sci Rep. 2017 Mar 8;7:44165. doi: 10.1038/srep44165 (PMC5341160; doi:10.1038/srep44165)
Supplement: Supplementary Information [file srep44165-s1.pdf]

Supplementary Information

A 3 base pair deletion in *TBX1* leads to reduced protein expression and transcriptional activity

Yuejuan Xu[1, 4*], Shaohai Fang[1*], Erge Zhang[1], Tian Pu [1], Ruixue Cao[1], Qihua Fu[2], Fen Li[3], Sun Chen[1], Kun Sun[1, 3], Rang Xu[1, 4#]

1 Department of Pediatric Cardiology, Xinhua Hospital, Affiliated to Shanghai Jiao Tong University School of Medicine, Shanghai 200092, China.

2 Medical Laboratory, Shanghai Children's Medical Center, Affiliated to Shanghai Jiaotong University School of Medicine, Shanghai 200127, China.

3 Department of Pediatric Cardiology, Shanghai Children's Medical Center, Affiliated to Shanghai Jiaotong University School of Medicine, Shanghai 200127, China.

4 Scientific Research Center, Xinhua Hospital, Affiliated to Shanghai Jiao Tong University School of Medicine, Shanghai 200092, China.

[#]corresponding author

Email: rangxu@shsmu.edu.cn

[*]These authors contributed equally to this work.

Legends of Supplementary Figures

Figure S1. Full-length western blots show the protein level of the mutant $TBX1^{102delK}$ and wild-type TBX1 in 3T3 cells. The results of 3 times transfection experiments show that the protein level of the mutant was reduced compared with the wild-type TBX1.

Figure S2. Full-length western blots show the protein level of the mutant $TBX1^{102delK}$ and wild-type TBX1 in C2C12 cells. The results of 3 times transfection experiments show that the protein level of the mutant was reduced compared with the wild-type TBX1.

Figure S3. Full-length image of electrophretic mobility shift assay to study DNA-binding activity of wild-type and mutant TBX1. In the condition of adding MG-132 to the culture medium (lane 1-4), both the wild-type and p.102delK mutant TBX1 proteins level are all increased (Figure 5 and S4). And DNA binding assay show that both the wild-type and mutant TBX1 could bind to DNA (lane 2 and 3). Specificity of the binding is confirmed by nuclear protein of HEK293T cell transfected with the pcDNA3.1(+) control vector (lane 1) and the addition of unlabled probe (100X) (lane 4). In the absence of MG-132 (lane 5-8), the specific binding bands aren't obvious. We thought that the less pronounced bands were due to the low proteins level of the wild-type and p.102delK mutant TBX1 in the absence of MG-132.

Figure S4. Full-length western blots show that proteasome inhibitor MG-132 increases both wild-type and mutant TBX1 protein level. C2C12 cells and 3T3 cells were transfected with equal concentrations of TBX1-encoding plasmids (the wild-type or the mutant 102delK) and treated with 20uM MG-132 or 10uM E-64 for 8 hours. The TBX1 levels were determined by Western blot (A: C2C12 cells; B: 3T3 cells). GAPDH was used as an internal control. n=3. Wt: wild-type; Mut: the $TBX1^{102delK}$ mutant; vehicle: DMSO.

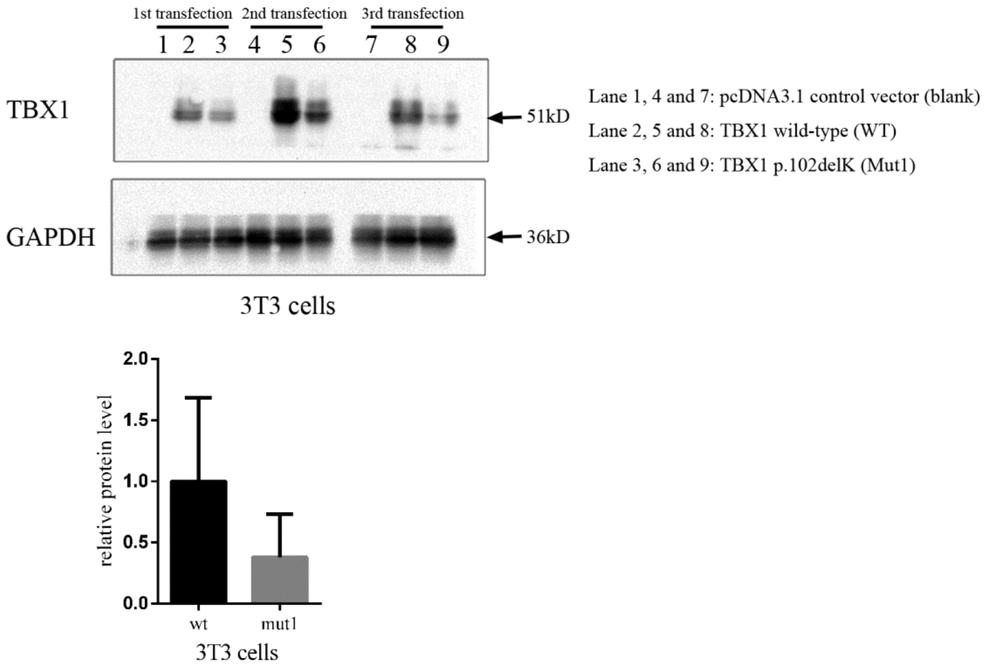

Figure S1

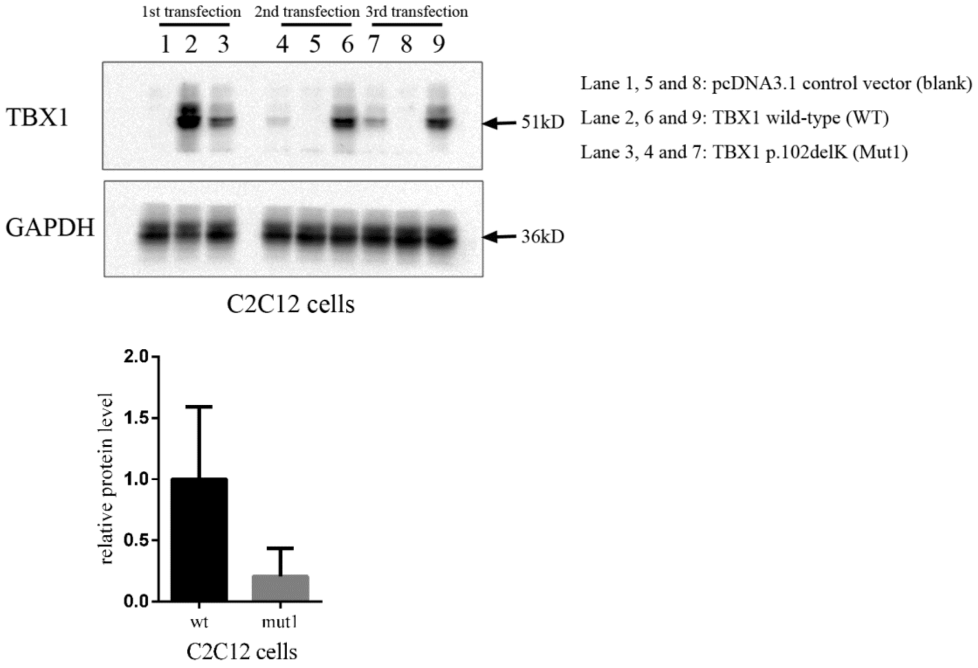

Figure S2

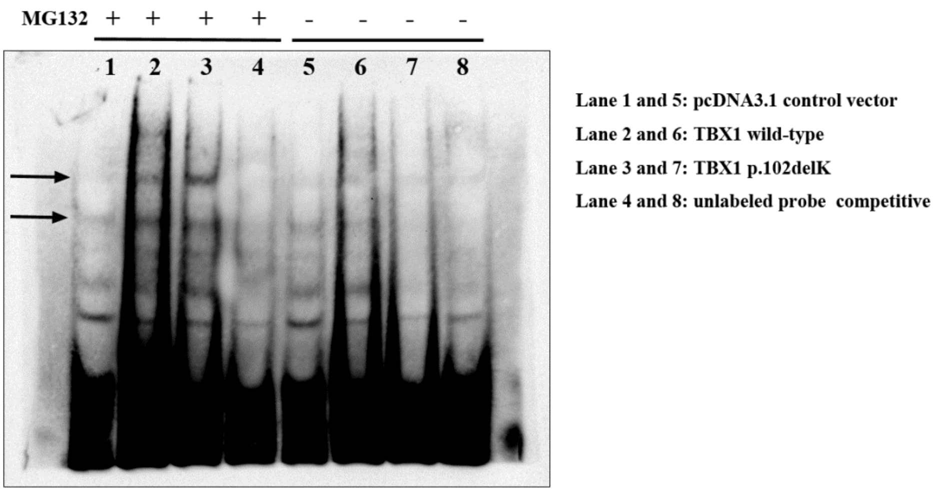

Figure S3

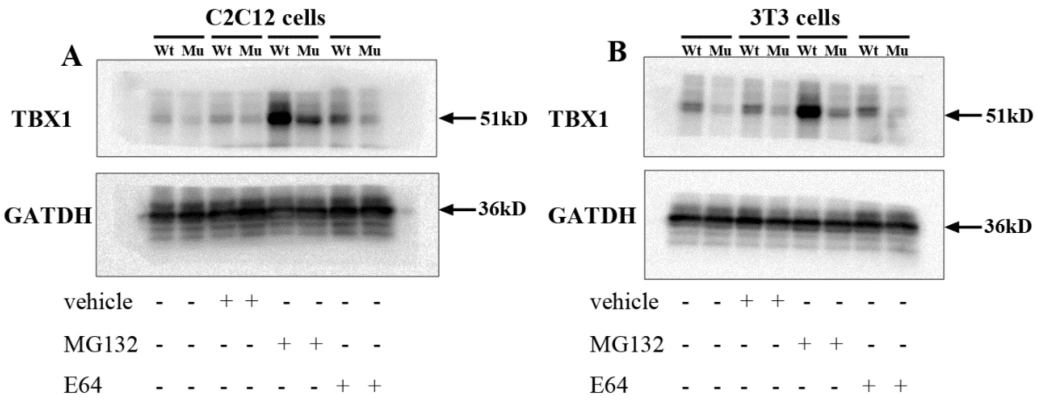

Figure S4
